# Transcriptome Variations in *Verticillium dahliae* in Response to Two Different Inorganic Nitrogen Sources

**DOI:** 10.3389/fmicb.2021.712701

**Published:** 2021-07-28

**Authors:** Chen Tang, Wenwen Li, Steven J. Klosterman, Yonglin Wang

**Affiliations:** ^1^Beijing Key Laboratory for Forest Pest Control, College of Forestry, Beijing Forestry University, Beijing, China; ^2^Agricultural Research Service, United States Department of Agriculture, Salinas, CA, United States

**Keywords:** *Verticillium dahliae*, nitrogen metabolism, transcriptome, nitrate assimilation, ammonium assimilation

## Abstract

The fungus *Verticillium dahliae* causes vascular wilt disease on hundreds of plant species. The main focus of the research to control this fungus has been aimed at infection processes such as penetration peg formation and effector secretion, but the ability of the fungus to acquire and utilize nutrients are often overlooked and may hold additional potential to formulate new disease control approaches. Little is known about the molecular mechanisms of nitrogen acquisition and assimilation processes in *V. dahliae*. In this present study, RNA sequencing and gene expression analysis were used to examine differentially expressed genes in response to the different nitrogen sources, nitrate and ammonium, in *V. dahliae*. A total of 3244 and 2528 differentially expressed genes were identified in response to nitrate and ammonium treatments, respectively. The data indicated nitrate metabolism requires additional energy input while ammonium metabolism is accompanied by reductions in particular cellular processes. Gene ontology and Kyoto Encyclopedia of Genes and Genomes pathway analyses of DEGs during nitrate metabolism revealed that many of the genes encoded those involved in protein biosynthetic and metabolic processes, especially ribosome and RNA polymerase biosynthesis, but also other processes including transport and organonitrogen compound metabolism. Analysis of DEGs in the ammonium treatment indicated that cell cycle, oxidoreductase, and certain metabolic activities were reduced. In addition, DEGs participating in the utilization of both nitrate and ammonium were related to L-serine biosynthesis, energy-dependent multidrug efflux pump activity, and glycerol transport. We further showed that the mutants of three differentially expressed transcription factors (*VdMcm1*, *VdHapX*, and *VDAG_08640*) exhibited abnormal phenotypes under nitrate and ammonium treatment compared with the wild type strain. Deletion of *VdMcm1* displayed slower growth when utilizing both nitrogen sources, while deletion of *VdHapX* and *VDAG_08640* only affected nitrate metabolism, inferring that nitrogen assimilation required regulation of bZIP transcription factor family and participation of cell cycle. Taken together, our findings illustrate the convergent and distinctive regulatory mechanisms between preferred (ammonium) and alternative nitrogen (nitrate) metabolism at the transcriptome level, leading to better understanding of inorganic nitrogen metabolism in *V. dahliae*.

## Introduction

Nitrogen is an indispensable element of biomolecules containing amino acids, nucleotides, and organic cofactors. In all living organisms, nitrogen metabolism requires subtle adjustments to balance metabolite synthesis with uptake from exogenous sources and to ensure survival during conditions of nitrogen starvation (Gouzy et al., 2014). In fungi, ammonium (NH_4_^+^) and glutamine act as preferred nitrogen sources which are prioritized in their utilization to promote fungal survival and fitness (Ries et al., 2018). In the absence of preferred nitrogen sources, alternative nitrogen sources such as nitrate (NO_3_^–^) may be utilized causing a switch from nitrogen-associated anabolism to catabolism (Tudzynski, 2014). Nitrogen metabolism plays key roles in the growth and development of plants and microbes, and can affect the fate of an interaction between plants and pathogens (Kusano et al., 2011; Gupta et al., 2013; Thalineau et al., 2016). Acquisition of nitrogen from the host tissues is pivotal to proper infection-related processes because nitrogen supports energy production, provides raw material for growth, and is essential in regulatory or signaling processes in fungal development (Deng et al., 2015).

In plants, nitrogen is transported from the roots to the shoot, mainly as inorganic nitrogen, amino acids, amides, and ureides (Divon et al., 2005). Ammonium and nitrate represent two of the predominant inorganic nitrogen sources that are absorbed by higher plants (Wang M. et al., 2019). The content of these two compounds are diverse in different plant tissues, indicating that the nitrogen sources available for a pathogen in the host plant are dependent on the tissue that is being colonized. Thus it is plausible that for a soilborne pathogen which invades roots and a foliar pathogen, there may be different mechanisms regulating nitrogen metabolism (Snoeijers et al., 2000). For those vascular pathogens that colonize the xylem, the nutrient deprivation for the pathogen may be extreme since the inside of xylem vessels is recognized as a nutritionally poor environment, although it allows efficient transport of water and soluble mineral nutrients from the root to the upper plant parts (Singh et al., 2010). With only minute amounts of solutes (Fisher, 2000), only a small minority of pathogens have adapted to the vascular system of xylem vessels as a major ecological niche in their disease cycles. In the foliar pathogen, *Magnaporthe oryzae*, nitrogen utilization, metabolism of some amino acids, and early infection in the nitrogen-poor confines of the apoplast were studied in depth, revealing that nitrogen sources have an impact on appressorium formation in *M. oryzae* (Froeliger et al., 1996; Wilson et al., 2010; Fernandez and Wilson, 2012; Fernandez et al., 2012; Marroquin-Guzman and Wilson, 2015). Nevertheless, knowledge of nitrogen metabolism of other phytopathogenic fungi is limited.

Vascular wilt diseases caused by pathogenic fungi that proliferate in the water-conducting xylem vessels cause typical symptoms including wilt or flaccidity of the leaves and sometimes death of the plant (Yadeta and Thomma, 2013). For growth, reproduction, and survival, vascular pathogens must readily acclimate to limited-nitrogen environments and efficiently acquire nitrogen resources essential for normal physiological activities in the xylem sap (Divon et al., 2005). Due to the specific niche of vascular pathogens within the xylem, it is difficult to characterize or quantify their adaptations to this environment. Thus, relatively little is known about their metabolism in response to limited nitrogen supplies in the xylem on the molecular and biochemical levels compared to foliar pathogens (Berne and Javornik, 2016). Consequently, it is of theoretical and practical importance to understand the molecular mechanisms of how vascular pathogens adapt to poor nutrition environments and metabolize nitrogen sources from plant hosts so as to design potentially novel control strategies to combat vascular wilt diseases.

Vascular wilt diseases known as Verticillium wilts are caused primarily by the fungal species *V. dahliae*, which infects over 200 plant species worldwide (Klosterman et al., 2009). Verticillium wilts result in yield losses of economically important crops and also affects numerous ornamental shrubs and trees (Klosterman et al., 2009). In China, *V. dahliae* causes high mortality rates of ornamental perennials like smoke trees (*Cotinus coggygria*), the leaves of which provide beautiful scenery at the Fragrant Hills Park in Beijing, China (Wang et al., 2013). Owing to the vascular habitat of the pathogen, no fungicides can provide remedial action once the plant is infected, and control of the pathogen in the field is also difficult since it survives for years in the soil (Klosterman et al., 2009). Though nitrogen metabolism plays indispensable roles in numerous aspects of fungal growth and virulence, there remains a number of questions on nitrogen usage that demand explanation. Relative to the numerous studies on infection structure formation and effectors in *V. dahliae*, the processes of nitrogen acquisition and assimilation in *V. dahliae* have not received adequate attention.

In previous studies, at least two genes in *V. dahliae* were implicated in nitrogen metabolism, including *VdCpc1* which was required for resistance to amino-acid starvation as well as infection and colonization (Timpner et al., 2013). Additionally *VdAtf1* serves as a novel transcription factor (TF) that regulates nitrogen assimilation and further links nitrogen metabolism with virulence (Tang et al., 2020b). Other TFs also take part in nitrogen metabolic processes indirectly (Tang et al., 2020a). However, questions still abound on the influence of nitrogen metabolism on various aspects of the *V. dahliae* disease cycle. The purpose of the current study was to understand how *V. dahliae* utilizes diverse inorganic nitrogen sources through transcriptome analysis, especially in a limited nitrogen environment. Our results demonstrate that alternative nitrogen metabolism is less preferred because of the costs associated with the expression of additional gene sets that may not directly influence nitrogen metabolism. In contrast, the preferred nitrogen source stimulated gene expression directly associated with nitrogen metabolism while restraining expression of those genes involved in unrelated cellular and metabolic processes. Results of the data analyses highlight the convergent and distinctive regulatory mechanisms between preferred and alternative nitrogen metabolism, providing an increased understanding of nitrogen metabolism in *V. dahliae*.

## Materials and Methods

### Fungal Strain and Culture Conditions

The wild type *V. dahliae* strain XS11 was isolated from a smoke tree in Fragrant Hills, Beijing (Wang et al., 2013) and was used for transcriptome profile analyses in this study. Mutants including Δ*VdMcm1*, Δ*VdHapX*, Δ*VDAG_08640* were obtained in our previous studies (Xiong et al., 2016; Fang et al., 2017; Wang et al., 2018). Conidial suspensions of all strains were stored long term at –80°C in 30% glycerol. All strains were initially grown on potato dextrose agar (PDA) plates (200 g potato, 20 g glucose, 15 g agar per liter) at room temperature.

For growth tests on TOR and PKA inhibitors, the XS11 strain was cultured for 10 days on complete medium (CM, 50 ml 20 nitrate salts, 1 ml 1000X trace elements, 10 g glucose, 2 g peptone, 1 g yeast extract, 1 g casamino acids, 1 ml vitamin solution per liter) and on defined minimal media containing 1% (w/v) glucose (1 l GMM, 1.52 g KH_2_PO_4_, 0.52 g KCl, 0.152 g MgSO_4_⋅7H_2_O, 3 μM thiamine HCl, 1.98 g glucose, 15 g agar per liter) with 10 mM of the two sole nitrogen sources nitrate (NO_3_^–^) and ammonium (NH_4_^+^) at 25^*o*^C, and with 10 μM rapamycin and 40 μM *N*-[2-(*p*-Bromocinnamylamino)ethyl]-5-isoquinolinesulfonamide ⋅ 2HCl hydrate (H-89). The colony diameter was measured after 10 dpi (days post inoculation). The inhibited hyphal growth was calculated using difference value of growth diameters of the XS11 strain when cultured on CM and on different nitrogen sources divided by growth diameters of it grown on CM, and those of the XS11 strain treated by rapamycin or H-89 were used the same method of calculation. All of the experiments were repeated three times.

For growth tests on single nitrogen sources, all strains were cultured for 10 days on CM and on GMM with 10 mM of the two sole nitrogen sources nitrate (NO_3_^–^) and ammonium (NH_4_^+^) at 25^*o*^C. The colony diameter was measured after 10 dpi. The diameters of irregular colonies were measured form multiple different directions and calculated the average value. The inhibited hyphal growth was calculated using difference value of growth diameters of the XS11 strain when cultured on CM and on different nitrogen sources divided by growth diameters of it grown on CM, and those of the mutants were used the same method of calculation. All of the experiments were repeated three times.

### RNA Extraction

The 1 ml conidial suspension (10^7^ spores/ml) of the XS11 strain was added into 100 ml liquid CM and shaken for 3 days. The fresh hyphae were collected by filtration from the liquid shake cultures using a single-layer of Miracloth (Millipore) and were washed by sterile water three times. Then the vegetative hyphae were switched to liquid GMM with 10 mM of each of the sole nitrogen sources, including nitrate (NO_3_^–^) or ammonium (NH_4_^+^) at 25°C for 24 h. The fresh hyphae were collected by filtration from the liquid shake cultures using a single-layer of Miracloth (Millipore) and frozen in liquid nitrogen immediately. All of the fungal samples were ground to a fine powder using a mortar and pestle in liquid nitrogen. Total RNA was extracted by TRIzol reagent (Invitrogen) and purified with a PureLink RNA minikit (Ambion) in accordance with the manufacturers’ instructions.

### RNA-Seq and Mapping

After checking the quantity and quality of RNA using a NanoPhotometer spectrophotometer (Implen) and an RNA Nano 6000 Assay Kit of the Agilent Bioanalyzer 2100 system (Agilent Technologies), high quality RNA samples were chosen for RNA-Seq analyses. Three biological replicates under each condition were sequenced by a DNBSEQ-T7 platform (Beijing Genomics Institute), yielding over 45 million reads per sample.

Sequence data filtering was performed using SOAPnuke software (Beijing Genomics Institute). Raw reads were processed using in-house Perl scripts and clean reads were obtained by removing adapter sequences and those reads with low quality and poly-N sequences from the raw data. These high quality data were used to estimate the abundance of each transcript using the *V. dahliae* strain VdLs.17 reference genome from the Broad Institute (Klosterman et al., 2011) and Hierarchical Indexing for Spliced Alignment of Transcripts (HISAT) software (Kim et al., 2015). The reads were aligned to the reference genome (Klosterman et al., 2011) using Burrows-Wheeler transformation (Li and Dewey, 2011) to count the read numbers mapped to each gene. The fragments per kilobase per transcript per million mapped reads (FPKM) values were calculated and used to estimate the effects of sequencing depth and gene length on the mapped read counts. Principal Component Analysis (PCA) analysis and Pearson correlation coefficient matrix were calculated by the princomp function and cor function in R software.

### Differential Gene Expression Analysis

DEseq2 (Love et al., 2014) was used to provide statistical analyses for determining differential expression in digital gene expression data. The sets of differentially expressed genes (DEGs) between each pair of libraries were determined by performing Chi-square tests with *P* < 0.05. Significantly expressed genes with a |log2(fold-change)| > 1 and Adjusted *P*-value < 0.05 were selected. The assembled transcripts in the final transcriptome were annotated by mapping them to several public databases.

Gene Ontology (GO) classification of identified DEGs in response to various nitrogen sources was conducted according to functional annotations of DEGs and the GO resource^1^. The DEGs were categorized in terms of biological process, molecular function, and cellular component categories in a GO enrichment analysis, using a GOseq R package (Young et al., 2010), which corrected for gene length bias. GO terms with corrected *P-*values < 0.05 were considered to be significantly enriched within the DEG sets. The corrected *P*-values were adjusted using the Benjamini and Hochberg method (FDR < 0.05) as the threshold for significant differential expression (Benjamini and Yekutieli, 2001). Based on the Kyoto Encyclopedia of Genes and Genomes (KEGG) annotation results, the DEGs were classified into biological pathways. The KEGG database (Hu et al., 2018) was used to identify enriched pathways by a two-tailed Fisher’s exact test to test the enrichment of the differentially expressed individual gene products against all those identified. Significant enrichment was determined at *P* < 0.05. These pathways were classified into hierarchical categories according to the KEGG website^2^. Heat maps of expression values were normalized and drawn by TBtools (Chen et al., 2020).

### Measurements of Glycerol Content

The mycelia of the XS11 strains were obtained after induction conditions and ground to a fine powder. Subsequently, the powder (0.1 g) of each was transferred to a 2-ml tube containing 1 ml glycerol extraction buffer (Applygen Technologies Inc.). After vortexing for 5 min, the tubes were centrifuged at 5000 *g* for 20 min, 10 μl of supernatant was mixed with 195 μl detection buffer (Applygen Technologies Inc.). After the mixture was incubated at 37^*o*^C for 20 min, the glycerol concentration was determined by a spectrophotometer (SpectraMax 190) at 550 nm. The experiment was repeated three times.

### Protein Extraction

Fungal samples were ground to a powder in liquid nitrogen and transferred to a 5 ml centrifuge tube. Four volumes of lysis buffer containing 10 mM dithiothreitol, 1% triton-100, and 2 mM ethylenediaminetetraacetic acid (EDTA) (Sigma) were added to the powder, followed by sonication three times on ice using a high intensity ultrasonic processor (Scientz). An equal volume of Tris-phenol was added. Debris were removed following centrifugation at 5000 × *g* at 4°C for 10 min. The protein precipitate was washed with methanol and acetone respectively and was redissolved in 8 M urea. The protein concentration was determined with a BCA kit (Protein Assay Kit) (Beyotime) according to the manufacturer’s instructions.

### Statistical Analysis

Data were expressed as mean value ± standard error of the mean. Statistical analyses were performed by ANOVA and Student’s *t-*test (SPSS 16.0). The *P-*value < 0.05 and <0.01 was considered statistically significant in this study.

### Availability of Data and Material

Raw sequences were deposited in the National Center for Biotechnology Information (NCBI) and can be accessed in the Short Read Archive (SRA) database^3^ under accession SRX11048827-SRX11048835 for XS11, XS11-NO_3_^–^, and XS11-NH_4_^+^ respectively.

## Results

### RNA-Seq Profiles of *Verticillium dahliae* in Response to Nitrate and Ammonium Treatments

Xylem vessels of plants allow efficient transport of some soluble nitrogen sources such as ammonium and nitrate from the root to the upper plant parts, which can be used by *V. dahliae* as nutrients. Nevertheless, it is a nitrogen-deficient environment, and *V. dahliae* must acclimate rapidly for survival. To explore transcriptomes of *V. dahliae* on various inorganic nitrogen sources, three biological replicates of the XS11 strain were harvested for transcriptome sequencing using RNA-seq for each condition (non-treated, 10 mM nitrate, and 10 mM ammonium). These nine RNA-seq datasets were sequenced using DNBSEQ-T7, and approximately 47 million reads were obtained for each of the libraries. By removing adaptor sequences and undesirable reads (ambiguous, low quality, and duplicated sequence reads), clean reads were generated from the nine libraries with Q30 > 85%, suggesting high quality for the present sequencing results (Table 1). The total number of clean reads per library ranged from 42.09 to 44.78 million, and clean bases ranged from 6.31 to 6.72 Gb. Clean reads from all libraries were aligned to annotated genes in the *V. dahliae* VdLs.17 genome (Klosterman et al., 2011), and the fragments per kilobase per transcript per million mapped reads (FPKM) value was calculated for each gene.

**TABLE 1:** RNA sequencing statistics.

Sample	Total raw reads (M)^a^	Total clean reads (M)^b^	Clean reads Q30 (%)^c^	Total mapping (%)^d^	Uniquely mapping (%)^e^
XS11_1	49.08	44.78	85.28	90.91	62.83
XS11_2	47.33	43.51	85.91	90.97	63.23
XS11_3	47.33	43.18	85.19	91.06	62.48
XS11-NO3_1	47.33	43.84	88.12	91.75	68.26
XS11-NO3_2	47.33	43.89	87.94	91.54	68.03
XS11-NO3_3	45.57	42.09	87.51	91.49	66.78
XS11-NH4_1	47.33	43.45	86.4	91.15	67.39
XS11-NH4_2	47.33	43.42	86.44	91.06	66.66
XS11-NH4_3	47.33	43.49	86.42	91.22	66.8

*^*a*^Number of reads generated from the BGISEQ-500.*

*^*b*^Raw data with low-quality reads, reads containing 3’ adaptors, and reads containing two or more N bases removed.*

*^*c*^Percentage of bases with a mass value > 30.*

*^*d*^Number of reads mapped to the reference genome within 2-bp mismatch.*

*^*e*^Uniquely mapped = the number of reads mapped to the reference genome with unique sequence location.*

**FIGURE 1:**
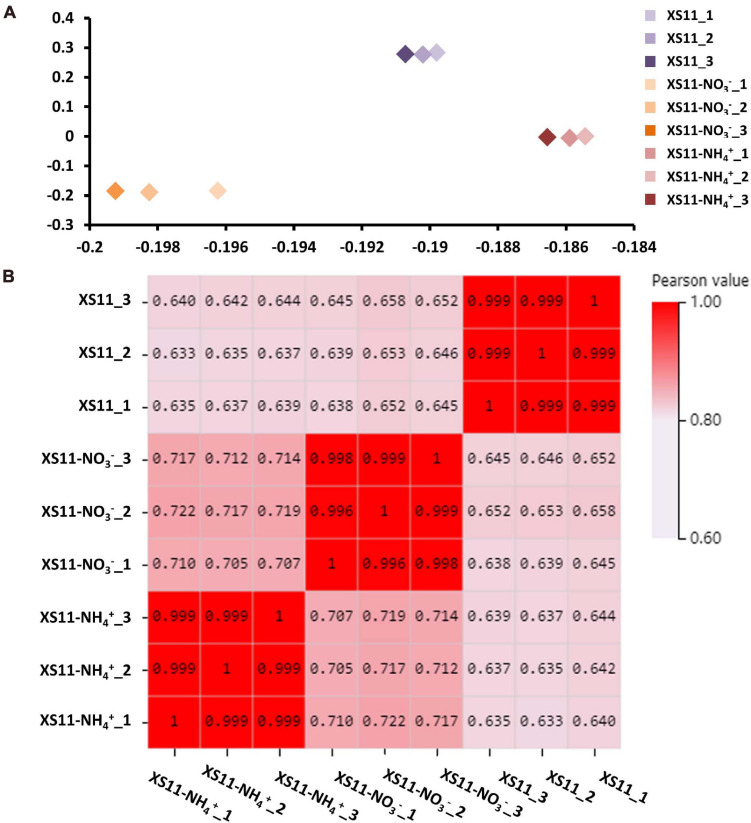
Repeatability of RNA-seq expression profiles in biological replicates in *Verticillium dahliae* in response to two different nitrogen sources. **(A)** Principle component analysis (PCA) of expression data about samples with or without treatments including 10 mM nitrate (NO_3_^–^) or 10 mM ammonium (NH_4_^+^) with three biological replicates. **(B)** Pearson’s correlation values (*P* < 0.01) non-clustering heatmap of nine samples displaying the correlation coefficient (*R*^2^).

The relationships among the nine samples were illustrated using a principal component analysis (PCA) performed with log2-normalized FPKM of all expressed genes. The samples from the same treatments were coordinated together, as expected, and the samples from the different treatments were clearly differentiated (Figure 1A). The Pearson’s correlation coefficients of paired samples were calculated using the FPKM values of all expressed genes for examination of the correlations between gene expression patterns among the nine libraries. All of the Pearson’s correlation coefficients were > 0.6. We also found the strong correlation among the samples of the same treatment (*R*^2^ = 0.996–1, *P* < 0.001), indicating a high level of reproducibility of the RNA-seq data (Figure 1B). Meanwhile, the samples obtained from different processing conditions yielded much lower correlation coefficient values. These results illustrated that samples in the same treatment groupings had consistency in their transcriptional changes and repeatability among the RNA-seq profiles.

### Identification of Differentially Expressed Genes (DEGs) in Response to Nitrate or Ammonium

**FIGURE 2:**
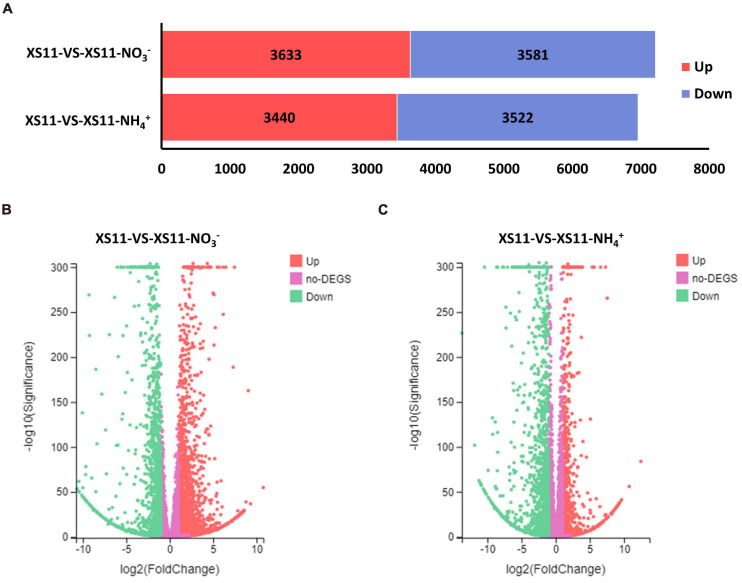
The number of differentially expressed genes (DEGs) in *Verticillium dahliae* in response to different nitrogen treatments. **(A)** Bar chart showing a different number of DEGs in the XS11 strain under different treatments in this study. The treatments included 10 mM nitrate (NO_3_^–^) or 10 mM ammonium (NH_4_^+^) amendments, or control. The red color represents gene upregulation while blue represents downregulation. **(B)** and **(C)**. Volcano plot showed the distribution of DEGs at | log2(fold-change) | > 1 and FDR *P* < 0.05 in the *V. dahliae* XS11 strain in response to 10 mM nitrate **(B)** or 10 mM ammonium **(C)** versus the control. The red point in the plot represents the upregulated DEGs with statistical significance, the green represents downregulated DEGs with statistical significance, and purple indicates DEGs without statistical significance.

Analyses of the DEGs collected may provide increased understanding into the regulatory changes in *V. dahliae* using different inorganic nitrogen sources. For this reason, we first identified all DEGs, and examined the major differences within these numbers between the nitrate or ammonium treatments. In total, expression of 8585 genes was altered between the two treatments. From a global overview, 7214 genes were affected under nitrate treatment including 3633 that were upregulated and 3581 that were downregulated in their expression (Figure 2A). Additionally, 6962 genes were influenced when ammonium served as the nitrogen source, with 3440 genes that were upregulated and 3522 that were downregulated. This finding suggested that *V. dahliae* responded by increased differential gene expression under nitrate treatment as compared to ammonium treatment.

Genes with insignificant differential expression values were filtered out, and DEGs (| log2(fold-change)| > 1 and Adjusted *P*-value < 0.05) were kept for further analyses. Based on the volcano plot analysis, 3244 genes were significantly differentially expressed in the XS11-NO_3_^–^ versus XS11 comparison, which represented nearly half of the affected genes in the nitrate treatment group (Figure 2B). Among these, 1840 DEGs were increased in their expression and 1404 were markedly reduced in their expression. While the XS11-NH_4_^+^ versus XS11 comparison revealed 922 upregulated and 1606 downregulated DEGs (Figure 2C). The up- and down-regulated DEGs were of about the same number in response to nitrate treatment, while the vast majority of DEGs were as downregulated in the ammonium treated group.

### Gene Ontology (GO) Analyses of DEGs

**FIGURE 3:**
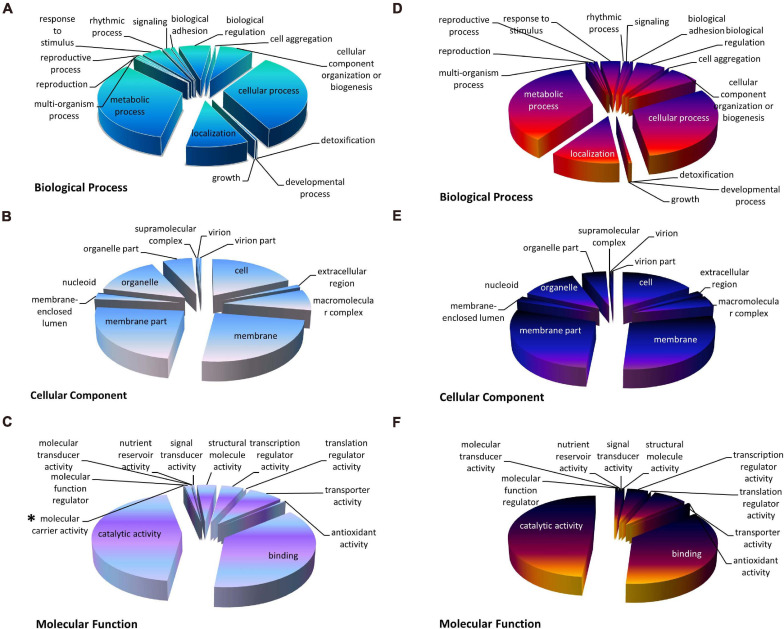
The Gene Ontology (GO) classification of DEGs under different nitrogen treatments in *Verticillium dahliae*. GO classification of identified DEGs based on their functional annotations in response to various nitrogen sources was conducted according to the GO resource (http://geneontology.org/). The treatments included 10 mM nitrate (NO_3_^–^) or 10 mM ammonium (NH_4_^+^) amendments, or control. **(A–C)** Indicates biological processes, cellular components, and molecular function under nitrate treatment, while **(D–F)** indicate biological processes, cellular components, and molecular function under ammonium treatment, respectively. *Indicates that the term appeared only once.

To understand the function of the DEGs uncovered in the nitrate and ammonium treated groups, the DEGs were distributed into three GO categories including biological process (BP), cellular component (CC), and molecular function (MF). Under both treatments, GO terms “catalytic activity,” “membrane,” and “metabolic process” accounted for the greatest number in MF, CC, and BP, respectively (Figure 3). DEGs of the XS11 strain treated by both nitrogen sources exhibited similar GO classifications and ratios. However, “molecular carrier activity” containing VDAG_04520 (encoding Cytochrome C Oxidase Copper Chaperone, COX17) VDAG_10000 (encoding exportin-1, XPO1) only appeared in the nitrate treatment group.

For further study, all DEGs were analyzed by GO enrichment. In the BP category, DEGs in response to the nitrate treatment were significantly enriched in 29 GO terms (Corrected *P*-value < 0.01), and the top five terms significantly enriched were macromolecule metabolic process (GO: 0043170); cellular nitrogen compound metabolic process (GO: 0034641); cellular macromolecule metabolic process (GO: 0044260); gene expression (GO: 0010467); and cellular macromolecule biosynthetic process (GO: 0034645) (Supplementary Table 1). Likewise, DEGs in response to ammonium treatment were significantly enriched in 59 GO terms (Corrected *P* value < 0.01), and the top five terms significantly enriched were organic cyclic compound metabolic process (GO: 1901360); cellular aromatic compound metabolic process (GO: 0006725); heterocycle metabolic process (GO: 0046483); nucleobase-containing compound metabolic process (GO: 0006139); and nucleic acid metabolic process (GO: 0090304). DEGs were gathered in different locations under different treatments in the CC category, non-membrane-bounded organelle, ribonucleoprotein complex, and ribosome for nitrate treatment whereas membrane and nucleus for ammonium treatment. GO enrichment of DEGs in the two treatments also showed similar and distinct differences in the MP category. Oxidoreductase activity (GO: 0016491) was the most significantly enriched term in response to both treatments, and DEGs of nitrate-treated XS11 were enriched in transporter activity (GO: 0005215) and transmembrane transporter activity (GO: 0022857) while DEGs of ammonium-treated XS11 were enriched in flavin adenine dinucleotide binding (GO: 0050660) (Supplementary Table 1).

### Kyoto Encyclopedia of Genes and Genomes (KEGG) Analyses of DEGs

**FIGURE 4:**
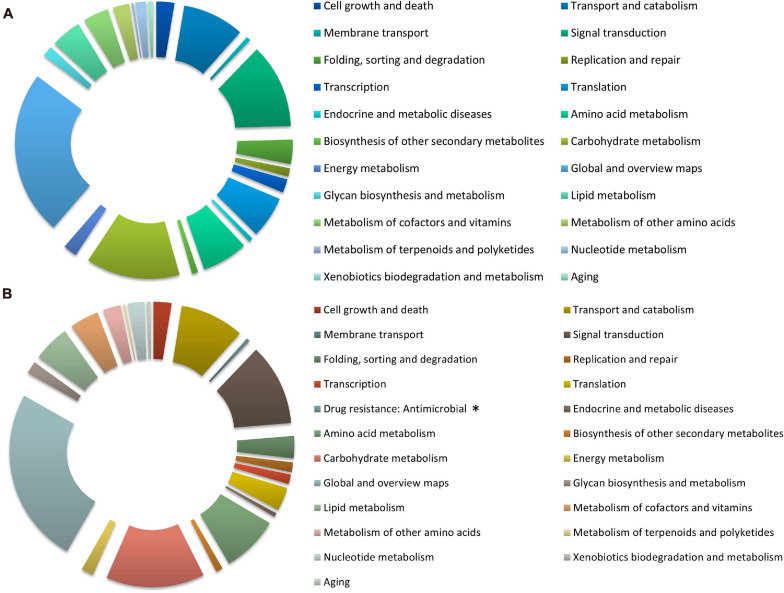
The Kyoto Encyclopedia of Genes and Genomes (KEGG) classification of DEGs under different nitrogen treatments in *Verticillium dahliae.* The pie charts exhibit KEGG classification of genes differentially expressed in *V. dahliae* strain XS11 in response to various nitrogen sources, including 10 mM nitrate (NO_3_^–^) or 10 mM ammonium (NH_4_^+^). **(A)** Represents XS11-NO_3_^–^ versus XS11 and **(B)** represents XS11-NH_4_^+^ versus XS11. ^∗^ Indicates that the pathway appeared only once. These pathways were classified into hierarchical categories according to the KEGG website (https://www.kegg.jp/kegg/kegg2.html).

The distribution of KEGG terms and their ratios in the six categories for each treatment are shown in Figure 4. A total of 3244 (nitrate) and 2528 (ammonium) genes were mapped to 22 and 23 KEGG pathways, respectively, and most of them were overlapping. The analysis showed that in the global and overview maps, carbohydrate metabolism, signal transduction, transport and catabolism, and amino acid metabolism were among the top five terms in all pathway categories under both treatment groups. “Drug resistance: Antimicrobial” pathway containing VDAG_05701 only appeared as enriched in response to ammonium treatment.

**FIGURE 5:**
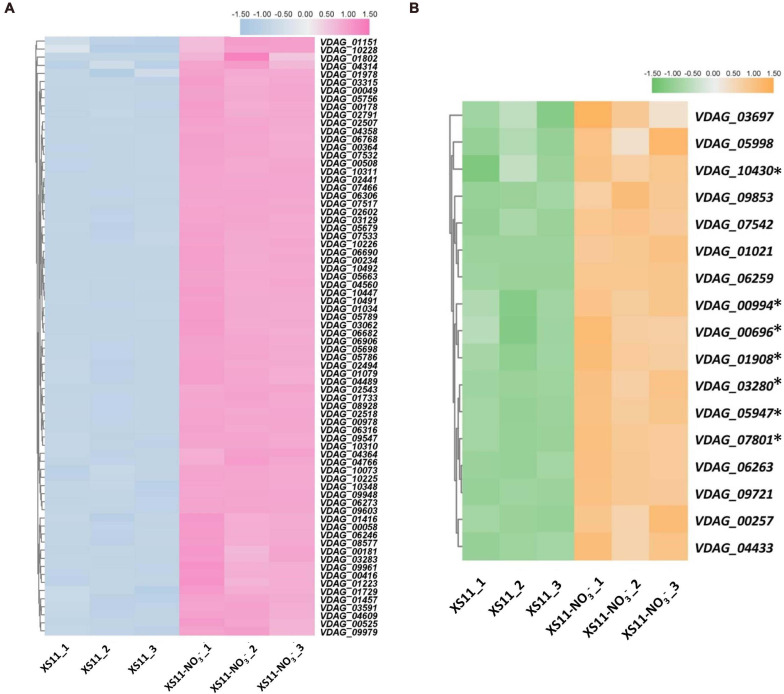
Heat maps of differentially expressed genes related to ribosome and RNA polymerase processes in response to nitrate as the sole nitrogen source in *Verticillium dahliae.* **(A)** Heat map showing the relative expression of genes related to ribosome in the *V. dahliae* XS11 strain using nitrate (NO_3_^–^) and in the control. TBtools was used to normalized data and generate heat map. The pink color indicates expression was relatively higher, while blue color indicates expression was relatively lower. White indicates that the gene expression between conditions analyzed was unchanged. **(B)** Heatmap of comparing expression of genes involved in RNA polymerase in nitrate metabolism. TBtools was used to normalized data and generate heat map. The orange color in the heat map indicates expression was relatively higher, while the green color in the heat map indicates expression was relatively lower. The white color indicates that the gene expression between conditions analyzed was unchanged. ^∗^Means the key genes in RNA polymerase.

Furthermore, KEGG enrichment was applied to identify pathways associating with the DEGs with diverse nitrogen utilization patterns (Supplementary Table 2). The KEGG enrichment revealed similar results to those of the GO enrichment, indicating that more genes related to ribosomes and transporters are upregulated in the nitrate treatment. Expression of genes related to terms ribosome (ko03010) and RNA polymerase (ko03020) were all highly upregulated (Figure 5), while ABC transporters (ko02010) were downregulated in response to the nitrate treatment. Enzymes involved in lipid metabolism, carbohydrate metabolism, and amino acid metabolism were abundant when assimilating and metabolizing ammonium, accounting for DEGs involved in these significantly enriched metabolic pathways.

**FIGURE 6:**
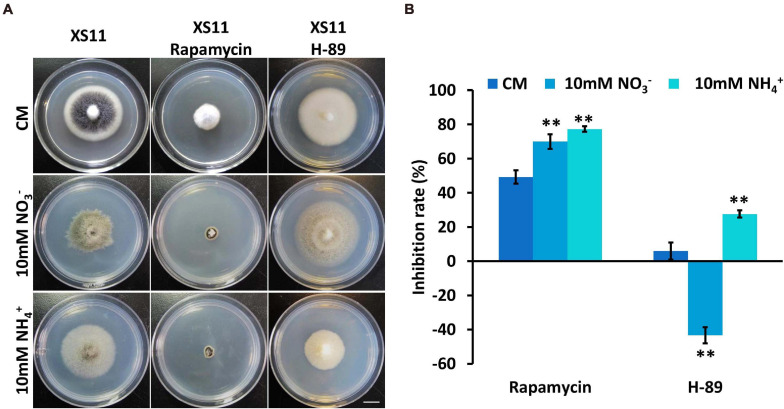
Responses of *Verticillium dahliae* to rapamycin and H-89 when utilizing two different nitrogen sources. **(A)** The XS11 strain was grown for 10 days on CM and on defined minimal media containing 1% (w/v) glucose (GMM) with 10 mM of the indicated sole nitrogen source including NO_3_^–^ and NH_4_^+^, and with 10 μM rapamycin and 40 μM *N*-[2-(*p*-Bromocinnamylamino)ethyl]-5-isoquinolinesulfonamide ⋅ 2HCl hydrate (H-89) at 25^*o*^C. Photographs were taken at 10 days post inoculation (dpi) of the media. Scale = 1.05 cm. **(B)** The chart illustrates the inhibition rates of the above colonies at 10 dpi. Error bars represent the standard deviation based on three independent replicates. Asterisks indicate significant differences (^∗∗^*P* < 0.01).

Considering the link between TOR and PKA pathways and ribosomal DNA transcription (Lippman and Broach, 2009), rapamycin was used to inhibit the TOR pathway and *N*-[2-(*p*-Bromocinnamylamino)ethyl]-5-isoquinolinesulfonamide ⋅ 2HCl hydrate (H-89) was used to inhibit Protein kinase A (PKA) when the XS11 strain metabolized nitrate and ammonium to examine the connection between ribosomal DNA transcription and nitrogen metabolism. Rapamycin led to higher inhibition rates under both treatments, while H-89 activated growth of the strain when it utilized nitrate (Figure 6). The expression of the key genes involved in TOR and PKA pathways were not changed significantly, inferring nitrogen metabolism impacted ribosomal DNA transcription through protein function rather than at the transcription level. Higher inhibition owing to restrained ribosomal DNA transcription may restrict nitrogen utilization of *V. dahliae*.

### Convergent and Distinctive Regulatory Mechanisms for Nitrate and Ammonium Metabolism

**FIGURE 7:**
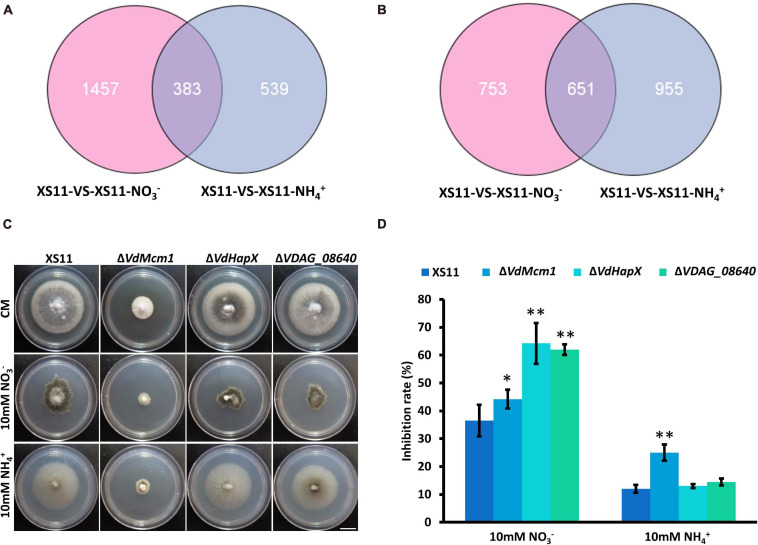
Upregulated and downregulated differentially expressed genes (DEGs) in response to two different nitrogen sources in *Verticillium dahliae.* **(A)** The Venn diagram shows the number of DEGs upregulated in the treatments of strain XS11 of *V. dahliae*. The treatments included 10 mM nitrate (NO3^–^) or 10 mM ammonium (NH4^+^) amendments, and control. The sum of numbers in each circle represents the total number of DEGs between the two samples; overlapping parts of the circle represent commonly expressed DEGs between the two treatments. Scale = 1.33 cm. **(B)** Venn diagram showing the number of DEGs downregulated in treatments. The sum of numbers in each circle represents the total number of DEGs between the two samples; overlapping parts of the circle represent commonly expressed DEGs between the two treatments. **(C)** All strains were grown for 10 days on CM and GMM with 10 mM of the indicated sole nitrogen source including NO_3_^–^ and NH_4_^+^ at 25°C. Photographs were taken at 10 dpi of the media. **(D)** The chart illustrates the inhibition rates of the above colonies at 10 dpi. Error bars represent the standard deviation based on three independent replicates. Asterisks indicate significant differences (^∗∗^*P* < 0.01; ^∗^*P* < 0.05).

To reveal the similarities and differences in gene expression during nitrate or ammonium utilization in *V. dahliae*, we applied Venn diagrams to profile the DEG distribution between XS11-NO_3_^–^ versus XS11 and XS11-NH_4_^+^ versus XS11. In the upregulated DEGs, 1457 DEGs were only significantly expressed in XS11-NO_3_^–^ versus XS11 comparison, which was nearly three times more than those only in XS11-NH_4_^+^ versus XS11, and 383 DEGs were highly upregulated in both groups (Figure 7A). However, the number of DEGs downregulated in XS11-NO_3_^–^ versus XS11 comparison was slightly lower than those downregulated in the XS11-NH_4_^+^ versus XS11 comparison (Figure 7B). The number of DEGs downregulated in both groups was nearly twice than that upregulated in both groups. VDAG_08640 was the top ten genes activated in both groups, and VDAG_05022 (*VdHapX*) was downregulated in response to both treatments. The mutant strains exhibited inhibited growth in nitrate utilization after deletion of the two genes, though they were similar to the XS11 strain when using ammonium (Figures 7C,D). VDAG_08640 and *VdHapX* might participate in nitrate metabolism other than ammonium utilization.

**FIGURE 8:**
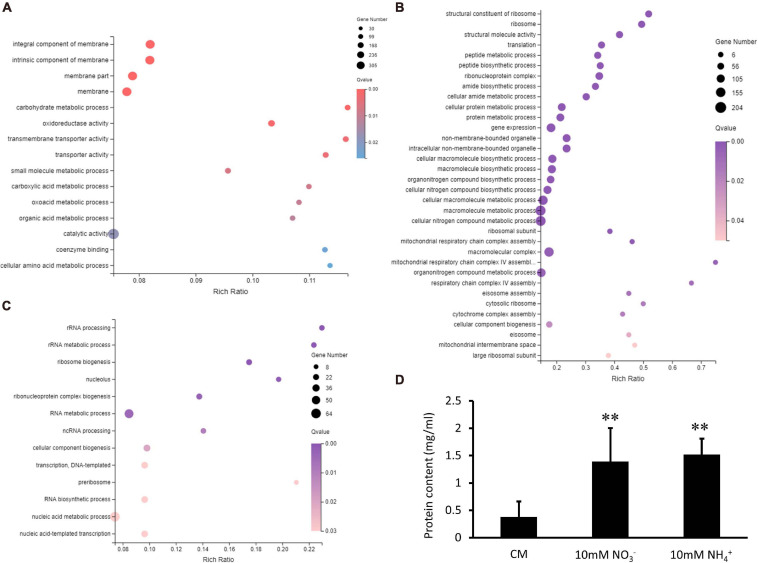
Enrichment analyses of differentially expressed genes (DEGs) in *Verticillium dahliae* in response to different nitrate sources. **(A)** A bubble chart showed DEGs depressed in both groups were enriched in 15 gene ontology (GO) terms. The size of the bubbles is proportional to the total number of DEGs. The *X*-axis represents the enrichment ratio and the *Y*-axis represents GO term. **(B)** A bubble chart showing DEGs whose expression was increased in nitrate utilization and enriched in 34 gene ontology (GO) terms by using a GOseq R package mentioned in section “Materials and Methods.” The size of the bubbles is proportional to the total number of DEGs. The *X*-axis indicates the enrichment ratio, and the *Y*-axis indicates the GO terms. **(C)** A bubble chart showing DEGs downregulated in nitrate utilization and enriched in 16 GO terms. The size of the bubbles is proportional to the total number of DEGs. The *X*-axis indicates enrichment ratio, and the *Y*-axis indicates GO terms. **(D)** Bar chart showing the protein content of the XS11 strain treated with 10 mM NO3^–^ or 10 mM NH4^+^ for 24 h. Error bars represent the standard deviation based on three independent replicates. Asterisks indicate significant differences (^∗∗^*P* < 0.01; ^∗^*P* < 0.05).

GO and KEGG enrichments were conducted for analyses of up and downregulated DEGs overlapped in the XS11-NO_3_^–^ versus XS11 and the XS11-NH_4_^+^ versus XS11 comparisons, respectively. There were no significant enrichments in upregulated DEGs and KEGG enrichment while DEGs which were downregulated in both groups were collected into 15 GO terms and more than one-third of these DEGs corresponded to the location term of membrane part. These also included terms of oxidoreductase activity (GO: 0016491); small molecule metabolic process (GO: 0044281); carbohydrate metabolic process (GO: 0005975); carboxylic acid metabolic process (GO: 0019752); oxoacid metabolic process (GO: 0043436); and alpha-amino acid metabolic process (GO: 1901605); transporter activity (GO: 0005215); transmembrane transporter activity (GO: 0022857); active transmembrane transporter activity (GO: 0022804); and copper ion transmembrane transporter activity (GO: 0005375) (Figure 8A). KEGG enrichment analysis of downregulated DEGs exhibited no significantly enriched pathway.

In the XS11-NO_3_^–^ versus XS11 group, upregulated and downregulated DEGs were significantly enriched in 34 and 16 GO terms, respectively. DEGs upregulated in the nitrate utilization group included those associated with terms non-membrane-bounded organelle and focused on nitrogen metabolism, including cellular nitrogen compound biosynthetic process (GO: 0044271), organonitrogen compound biosynthetic process (GO: 1901566); organonitrogen compound metabolic process (GO: 1901564); relevant processes of gene transcription and translation including gene expression (GO: 0010467); translation (GO: 0006412); ribosome biosynthesis such as ribonucleoprotein complex (GO: 1990904); ribosome (GO: 0005840); structural constituent of ribosome (GO: 0003735); peptide and protein biosynthetic and metabolic processes such as peptide metabolic process (GO: 0006518); peptide biosynthetic process (GO: 0043043); cellular protein metabolic process (GO: 0044267); protein metabolic process (GO: 0019538); other cellular process containing amide biosynthetic process (GO: 0043604); and cellular amide metabolic process (GO: 0043603) (Figure 8B). Additionally, KEGG enrichment analysis indicated one strikingly enriched pathway of ribosome (ko03010), which was consistent with the GO enrichment of upregulated DEGs. DEGs downregulated in this group were related to functions in binding ions and anions; adenyl nucleotide; adenyl ribonucleotide; ATP; GTPase; signal transduction such as Ras protein signal and small GTPase mediated signal transduction; active transmembrane transporter activity; and cell communication (Figure 8C). But KEGG enrichment analysis showed no significantly enriched pathway. *V. dahliae* activated numerous genes participating in protein biosynthesis when utilizing nitrate, and protein accumulated more in nitrate-treated XS11 vs. the ammonium-treated strain XS11 (Figure 8D).

**FIGURE 9:**
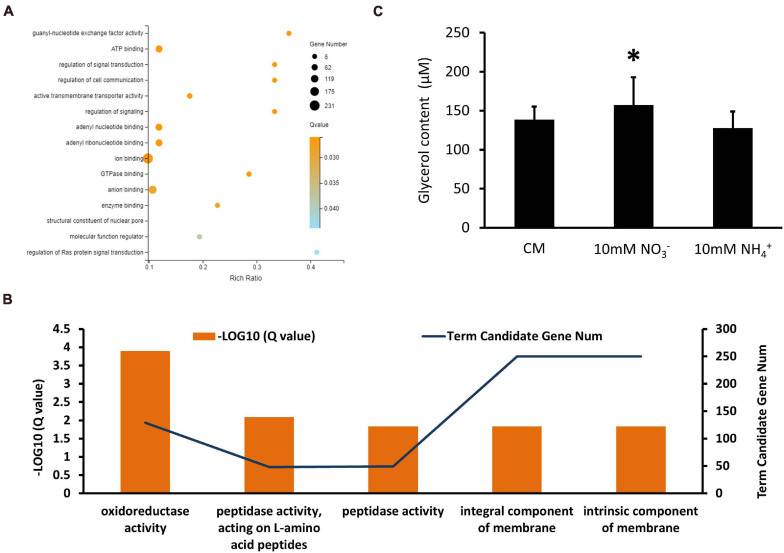
Enrichment analyses of differentially expressed genes (DEGs) in *Verticillium dahliae* in response to ammonium sources. **(A)** A bubble chart showing DEGs upregulated during ammonium utilization and enriched in 13 GO terms. The size of the bubbles is proportional to the total number of DEGs. The *X*-axis indicates enrichment ratio and the *Y*-axis indicates the GO terms. **(B)** A linear graph showing DEGs downregulated in ammonium utilization and enriched in five GO terms. **(C)** The bar chart showed glycerol content of the XS11 strain treated with 10 mM NO_3_^–^ or 10 mM NH_4_^+^ for 24 h. Error bars represent the standard deviation based on three independent replicates. Asterisks indicate significant differences (^∗^*P* < 0.05).

In the XS11-NH_4_^+^ versus XS11 group, upregulated and downregulated DEGs were significantly enriched in 13 and 5 GO terms, respectively. Among the upregulated DEGs, we identified a substantial number of those associated with RNA processes containing RNA biosynthetic process (GO: 0032774); RNA metabolic process (GO: 0016070); ncRNA processing (GO: 0034470); rRNA processing (GO: 0006364); and rRNA metabolic process (GO: 0016072); transcription such as transcription, DNA-templated (GO: 0006351); and nucleic acid-templated transcription (GO: 0097659); nuclear and ribosome including nucleolus (GO: 0005730); nucleic acid metabolic process (GO: 0090304); ribonucleoprotein complex biogenesis (GO: 0022613); preribosome (GO: 0030684); and ribosome biogenesis (GO: 0042254) (Figure 9A). Most of these were located in the compartment of the nucleolus. KEGG enrichment analysis showed no significantly enriched pathway in upregulated DEGs. Moreover, downregulated DEGs mostly localization as integral component of membrane and were enriched in oxidoreductase and peptidase activities (Figure 9B). Considering activation of genes involved in ribosome biogenesis and depression of those related to peptidase activity, we measured protein content when using ammonium, showing obvious protein accumulation (Figure 8D). KEGG enrichment analysis displayed pathways related to cell cycle (ko04111) and metabolism relating to lipid metabolism (ko00565); glycerophospholipid metabolism (ko00564); pyrimidine metabolism (ko00240); and glycine, serine, and threonine metabolism (ko00260). *VdMcm1* (*VDAG_01770*) is a conserved transcription factor regulating the cell cycle, so the Δ*VdMcm1* strain was tested here to ascertain its role in nitrogen utilization. Its inhibition rate was higher than the XS11 strain, which meaning *VdMcm1* takes part in regulation of nitrogen metabolism (Figures 7C,D). Genes involved in glycerophospholipid metabolism were depressed during ammonium utilization, which might cause perturbations in the glycerol content in hyphae. However, we found that ammonium treatment did not change glycerol content, while nitrate treatment caused its increase (Figure 9C). Our data showed that expression of VDAG_05602 (glycerol kinase, GK) was depressed in response to both treatments and the expression of VDAG_00445 (triacylglycerol lipase, TGL) was upregulated only when treated with nitrate, which might explain the differences in glycerol content in different nitrogen sources.

## Discussion

Nitrogen availability plays an essential role in growth, basic metabolic processes, and even plant-pathogen interactions for fungal pathogens (Kusano et al., 2011; Gupta et al., 2013; Thalineau et al., 2016). For acquisition and metabolism of different nitrogen sources, fungal pathogens have developed sophisticated mechanisms. There is a prioritization to use preferred nitrogen sources while repressing the utilization of alternative nitrogen sources, but utilizing alternative nitrogen sources causes switching from nitrogen-associated anabolism to catabolism (Tudzynski, 2014). Transcriptome analyses in this study showed clear similarities and differences between gene expression patterns in response to preferred or alternative nitrogen source utilization in *V. dahliae*. The higher numbers of different DEGs in the nitrate (NO_3_^–^) treatment versus the ammonium (NH_4_^+^) treatment suggested that alternative nitrogen sources require additional energy in metabolism, while preferred nitrogen sources such as ammonium are assimilated and metabolized in association with repression of certain cellular processes.

### Variation of Gene Expression in *V. dahliae* in Response to Preferred or Alternative Nitrogen Sources

Our previous study revealed that *V. dahliae* grew more slowly when nitrate was supplied as the sole nitrogen source vs. ammonium (Tang et al., 2020b). Herein, these transcriptome data provide evidence that may explain the differences in growth in response to either nitrogen or ammonium as the sole nitrogen source. As a whole, the two nitrogen metabolism affects the expression of genes with different functions and at different cellular sites or compartments. Nitrate utilization regulates genes located at ribosomes and non-membrane-bounded organelles and takes part in macromolecule and cellular nitrogen compound metabolic processes, gene expression, and transporter activity. However, ammonium utilization changes expression of genes located in membrane and nucleus and participates in some organic compound and nucleic acid metabolic process. During the utilization of nitrate shifts in metabolic processes are evident, and some these may be undesirable, but the choices of different compounds may be limited for survival. Additionally, the expression of genes involved in cellular organonitrogen compound biosynthetic and metabolic processes rose in response to greater extent during nitrate metabolism treatment rather than ammonium metabolism, suggesting that these genes are activated in response to alternative nitrogen metabolism.

Nitrate and ammonium metabolic processes employ different regulatory mechanisms despite their similar cellular processes. For example, among all upregulated DEGs in response to nitrate and ammonium the majority were enriched in transcriptional and translational processes, indicating the requirement by the pathogen to increase gene expression and protein translation to utilize nitrogen sources, which was evident by the accumulated protein content we observed in the nitrogen-treatment. The predicted locations of these products differed by organelle part: those in the nitrate treatment were localized in the ribosomal subunit and mitochondrial intermembrane space while those upregulated DEGs from the ammonium were mostly localized in nucleolus. These upregulated sets also have a bias toward distinct processes. The nitrate treatment correlated with increased numbers of DEGs associated with ribosome biosynthesis as well as protein biosynthetic and metabolic processes, while ammonium treatment resulted in upregulated DEGs that favored RNA and transcription related processes.

Ribosomes are primary consumers of cell resources and energy, thus accurate control of their biogenesis is fundamental for the maintenance of cellular growth. The transcription of ribosomal DNA is a key step in ribosome biosynthesis and a major biosynthetic and energy consuming process, which is tightly coupled to the availability of growth factors, nutrients and energy (Kusnadi et al., 2015). In a previous study, inhibition of mTORC1 with rapamycin led to reduced ribosomal DNA transcription, and it might related to amino acid availability (James and Zomerdijk, 2004; Hannan et al., 2013). Ribosome biosynthesis also depends on the balance between the economics of protein production and cell growth (Slavov et al., 2015). Pathogens may actively downregulate the expression of ribosomal genes to inhibit the translation system for efficient expression and translation of genes related to stress response under stress conditions (Spriggs et al., 2010). When confronted with nitrogen limitation, 17 selective ribosomal proteins were upregulated for activation of reserve translational capacity, revealing large reserves in metabolic and translational capacities of yeast (Yu et al., 2020). Rapamycin reduced hyphae growth when utilizing the two nitrogen sources herein, suggesting that the metabolism of the alternative nitrogen source requires normal ribosomal DNA transcription regulated by TOR, while the same process during ammonium utilization may be regulated by amino acid metabolism. PKA can also activate genes for ribosomal biogenesis (Lippman and Broach, 2009), however, inhibition of PKA led to higher growth rate during utilizing nitrate. DEGs related to signal transduction such as Ras protein signal and small GTPase mediated signal transduction were downregulated in nitrate utilization, while expression of genes encoding PKA did not changed significantly. Inhibition of PKA caused a corollary change in signal transduction related genes that matched those associated with PKA activity, potentially facilitating growth. It may be inferred that there was not enough ATP for PKA when nitrate was utilized, causing inhibition of growth.

Over-accumulation of glycerol in the cell may lead to various morphological defects and ultimately growth inhibition, though its normal accumulation is a cellular adaptation to high osmotic stress and salt stress (Randhawa et al., 2019). Fungi must maintain glycerol levels within limits suitable for growth, and tightly control its production and suppression (Kayingo et al., 2001). Glycerol uptake is also an important physiological mechanisms associated with glycerol utilization and osmoregulation (Ji et al., 2018). Stl1, a sugar transporter contributing to glycerol import, was first identified in *S. cerevisiae* to be involved in active glycerol uptake (Ferreira et al., 2005). It is also involved in osmotic response in *S. cerevisiae* and *C. albicans* (Kayingo et al., 2009; Bai et al., 2015). Stl1 was expressed in a complex manner by carbon source, during growth on non-fermentable carbon sources (Kayingo et al., 2009). The data herein revealed that the Stl1 homologs (VDAG_00764 and VDAG_05113) were evidently downregulated in response to either of the two nitrogen sources, presumably in order to remodel the glycerol balance because intense metabolic activity may consume glycerol as carbon source, and further inferring a connection between nitrogen and carbon metabolism. GK (glycerol kinase) can phosphorylate glycerol to glycerol-3-phosphate (G3P) for glycerol assimilation (Wilk et al., 2020). TGL (triacylglycerol lipase) can gradually hydrolyze triglycerides into glycerol and fatty acids (Wadsater et al., 2014). Herein, glycerol was accumulated because of activated TGL during nitrate utilization, though both nitrogen assimilations caused downregulated Stl1 and GK.

The regulation of the expression of two bZIP transcription factors in response to the different nitrogen sources was notable in these experiments. There was a striking upregulation of VDAG_08640, a bZIP transcription factor in response to both nitrogen sources. Previous work also revealed that this particular gene was differentially expressed during microsclerotia development, but does not regulate microsclerotia formation, the sensitivity to hydrogen peroxide, and virulence, hence was not considered to be indispensable in *V. dahliae* (Fang et al., 2017). Deletion of this gene caused an affect on nitrate utilization, suggesting a role of VDAG_08640 in nitrate metabolism. Nevertheless, involvement of the bZIP transcription factor in nitrogen metabolism is novel and requires further study. *VdHapX* is also a bZIP transcription factor contributing to adaptation to iron starvation and excesses, hydrogen peroxide detoxification, microsclerotium formation and virulence in *V. dahliae* (Wang et al., 2018). In *Fusarium graminearum*, the transcription factor cascade FgAreA-HapX link iron homeostasis to nitrogen metabolism: reduced activity of cytosolic Fe–S proteins nitrite reductase causes high expression of FgHapX via activating transcription factor FgAreA, then FgHapX suppresses another iron regulator FgSreA to maintain iron homeostasis (Wang Z. et al., 2019). Here, the expression of *VdHapX* was depressed in response both two nitrogen assimilation, while its knockout led to a clear growth defect during nitrate utilization. Potentially, deletion of *VdHapX* affected iron homeostasis, reducing the activity of nitrite reductase which is a key enzyme in nitrate assimilation and finally caused decreased growth during utilizing nitrate. However, nitrite reductase is dispensable in ammonium assimilation, thus deletion of *VdHapX* failed to impact this process. Its decreased expression may be related to *V. dahliae* placing increased energy into nitrogen utilization and depression of other metabolic processes for survival in a changing environment.

### Overlap Between Responses to Different Inorganic Nitrogen Sources in *V. dahliae*

Although there were various differences in DEGs associated with different metabolic pathways when supplied with preferred and alternative nitrogen sources in *V. dahliae*, some regulatory mechanisms are similar in both metabolic processes. The cell cycle, involving a series of complex cellular events, is vital to morphological changes related to infectious growth or infection-related morphogenesis in some fungal pathogens (Sendinc et al., 2015; Jiang et al., 2016). The cell cycle machinery and metabolism are interconnected. The initiation of cell cycle critically depends on the availability of metabolites, and it maintains cell survival and proliferation by regulating metabolic networks (Kaplon et al., 2015). In the absence of glucose, for example, cells may be arrested at the G1/S phase transition, demonstrating how carbon metabolism impacts cell cycle (Pardee, 1974). Herein, genes associated with cell cycle such as *VdMcm1* were downregulated during ammonium assimilation, inferring a link between nitrogen metabolism and the cell cycle. Deletion of *VdMcm1* resulted in significant reduction of hyphal growth (Xiong et al., 2016), which may explain the reduced growth when cultured on ammonium medium. The expression of VDAG_08640 was obviously reduced in the *VdMcm1* deletion mutant (Xiong et al., 2016), which inferred that VdMcm1 regulates nitrate utilization via affecting expression of VDAG_08640.

Expression of the gene encoding D-3-phosphoglycerate dehydrogenase (PGDH) (VDAG_01596), the committal enzyme in L-serine biosynthesis (Grant, 2018), was observably reduced in both conditions in which nitrate and ammonium were independently supplied as nitrogen sources. L-Serine is a crucial amino acid because it serves as a precursor the production of vital metabolites. The most-well-studied PGDHs are bacterial, primarily from *Escherichia coli* and *Mycobacterium tuberculosis*, showing the catalytic activity of PGDH and L-serine form a feedback loop and it is involved in bacterial motility, adherence and virulence (Zhao and Winkler, 1996; Peters-Wendisch et al., 2002; Yasuda et al., 2017). In plants, PGDH plays a vital function in plant development and metabolism, and participates in some pathways linking carbon and nitrogen metabolism and in maintaining cellular redox and energy levels in stress conditions (Igamberdiev and Kleczkowski, 2018). The ABC (ATP-binding cassette) transporter family, involved in resistance to azole antifungal agents, has been extensively documented (Coste et al., 2004). In *C. albicans*, *Cdr1* (*Candida* drug resistance) from the family of ABC transporters, a gene most often associated with an energy-dependent multidrug efflux pump, is the principal mediator of resistance to azoles due to transport phenomena (Sanglard et al., 1995). The efflux activity of *Cdr1* pumps is energy-dependent (Thomas et al., 2013). In our study, *Cdr1* homolog (VDAG_05293) expression was remarkably reduced when *V. dahliae* assimilated both nitrate and ammonium sources, which may indicate that the pathogen preserve energy to metabolize either of the supplied nitrogen sources.

## Conclusion

Based on the results presented herein, it is clear that metabolism of different nitrogen sources in *V. dahliae* triggers major differences in gene transcription. In this study, we highlighted the convergent and distinctive regulatory mechanisms between preferred and alternative nitrogen metabolism in *V. dahliae*. RNA-Seq analyses suggested that assimilation and metabolism of both nitrogen sources intensively impact many aspects of the oxidoreductase activity, transporter activity, and metabolic processes. However, the expression analyses also indicate that nitrate and ammonium metabolism are under different regulatory control mechanisms even though there were clearly also increases in transcription and translation processes when either was used as the sole nitrogen source. During utilization of nitrate, the expression analyses suggest that ribosome biosynthesis and protein translation are activated but signal transduction and communication are reduced. Additionally, gene expression associated with cellular nitrogen compound biosynthetic and metabolic processes increased in response to nitrate rather than ammonium as the sole nitrogen source, and therefore preferred nitrogen metabolism is apparently more efficient and less energy intensive. Further, according to our results, *V. dahliae* inhibits some cellular processes such as the cell cycle as well as other metabolic process during assimilation and metabolism of ammonium. Glycerol and protein content measurements verified some of these changes in this study, and bZIP transcription factors play an important role in nitrogen metabolism. This study provides useful information into our understanding of how *V. dahliae* utilizes two different nitrogen sources which may provide insights into alternative disease control measures.

## Data Availability Statement

Raw Illumina sequences were deposited in the National Center for Biotechnology Information (NCBI) and can be accessed in the Short Read Archive (SRA) database (http://trace.ncbi.nlm.nih.gov/Traces/sra/) under accession SRX11048827–SRX11048835 for XS11, XS11- NO_3_^–^, and XS11-NH4^+^ respectively.

## Author Contributions

YW and CT conceived the experiments. CT and WL performed the experiments. CT analyzed the data. CT, SK, and YW wrote the manuscript. All authors read and approved the manuscript.

## Conflict of Interest

The authors declare that the research was conducted in the absence of any commercial or financial relationships that could be construed as a potential conflict of interest.

## Publisher’s Note

All claims expressed in this article are solely those of the authors and do not necessarily represent those of their affiliated organizations, or those of the publisher, the editors and the reviewers. Any product that may be evaluated in this article, or claim that may be made by its manufacturer, is not guaranteed or endorsed by the publisher.

## Footnotes

1
http://geneontology.org/

2
https://www.kegg.jp/kegg/kegg2.html

3
http://trace.ncbi.nlm.nih.gov/Traces/sra/

## Supplementary Material

Supplementary Table 1 Enrichment terms of significantly differentially expressed genes (corrected *P*-value < 0.01).

Supplementary Table 2 Enrichment terms of significantly differentially expressed genes (corrected *P*-value < 0.05).
