# Supplementary material for: Transcriptome Variations in Verticillium dahliae in Response to Two Different Inorganic Nitrogen Sources
Source: Front Microbiol. 2021 Jul 28;12:712701. doi: 10.3389/fmicb.2021.712701 (PMC8355529; doi:10.3389/fmicb.2021.712701)
Supplement: Supplementary Table 1 — Enrichment terms of significantly differentially expressed genes (corrected P-value < 0.01). [file Table_1.DOCX]

**Table S1.** **Enrichment terms of significantly differentially expressed genes (corrected *P* value < 0.01)**

| **Functional categories** | **GO Term** | **Number of DEGs** | **Corrected *P* value** |
| --- | --- | --- | --- |
| **Biological_process** |  |  |  |
| XS11 VS XS11-NO_3_^-^ |  |  |  |
| GO:0006412 | translation | 86 | 1.93E-15 |
| GO:0043043 | peptide biosynthetic process | 86 | 4.01E-15 |
| GO:0006518 | peptide metabolic process | 89 | 5.81E-15 |
| GO:0043604 | amide biosynthetic process | 88 | 4.17E-14 |
| GO:0043603 | cellular amide metabolic process | 93 | 6.64E-12 |
| GO:0010467 | gene expression | 175 | 5.97E-07 |
| GO:0044267 | cellular protein metabolic process | 120 | 5.73E-06 |
| GO:0019538 | protein metabolic process | 121 | 1.63E-05 |
| GO:0034645 | cellular macromolecule biosynthetic process | 146 | 2.39E-05 |
| GO:0009059 | macromolecule biosynthetic process | 146 | 3.98E-05 |
| GO:0009311 | oligosaccharide metabolic process | 9 | 0.00034951 |
| GO:0044271 | cellular nitrogen compound biosynthetic process | 144 | 0.003140265 |
| GO:0005991 | trehalose metabolic process | 7 | 0.003255326 |
| GO:0005984 | disaccharide metabolic process | 7 | 0.003255326 |
| GO:0044260 | cellular macromolecule metabolic process | 202 | 0.006579685 |
| GO:0043170 | macromolecule metabolic process | 244 | 0.007905802 |
| XS11 VS XS11-NH_4_^+^ |  |  |  |
| GO:0042254 | ribosome biogenesis | 41 | 1.53E-10 |
| GO:0006364 | rRNA processing | 29 | 5.34E-09 |
| GO:0016072 | rRNA metabolic process | 29 | 8.77E-09 |
| GO:0022613 | ribonucleoprotein complex biogenesis | 43 | 6.69E-08 |
| GO:0034470 | ncRNA processing | 36 | 4.83E-07 |
| GO:0090304 | nucleic acid metabolic process | 126 | 3.35E-05 |
| GO:0009141 | nucleoside triphosphate metabolic process | 21 | 5.33E-05 |
| GO:0009145 | purine nucleoside triphosphate biosynthetic process | 15 | 8.19E-05 |
| GO:0009206 | purine ribonucleoside triphosphate biosynthetic process | 15 | 8.19E-05 |
| GO:0006396 | RNA processing | 51 | 0.000121108 |
| GO:0006754 | ATP biosynthetic process | 14 | 0.000239143 |
| GO:0009142 | nucleoside triphosphate biosynthetic process | 15 | 0.000239143 |
| GO:0009201 | ribonucleoside triphosphate biosynthetic process | 15 | 0.000239143 |
| GO:0046034 | ATP metabolic process | 17 | 0.000239143 |
| GO:0005975 | carbohydrate metabolic process | 81 | 0.000288142 |
| GO:0009144 | purine nucleoside triphosphate metabolic process | 18 | 0.000318317 |
| GO:0009205 | purine ribonucleoside triphosphate metabolic process | 18 | 0.000318317 |
| GO:0044085 | cellular component biogenesis | 57 | 0.000770514 |
| GO:0009199 | ribonucleoside triphosphate metabolic process | 18 | 0.000783143 |
| GO:0016070 | RNA metabolic process | 97 | 0.000940695 |
| GO:0009126 | purine nucleoside monophosphate metabolic process | 19 | 0.001540807 |
| GO:0009167 | purine ribonucleoside monophosphate metabolic process | 19 | 0.001540807 |
| GO:0034660 | ncRNA metabolic process | 37 | 0.001679522 |
| GO:0009127 | purine nucleoside monophosphate biosynthetic process | 16 | 0.002504134 |
| GO:0009168 | purine ribonucleoside monophosphate biosynthetic process | 16 | 0.002504134 |
| GO:0017144 | drug metabolic process | 37 | 0.002504134 |
| GO:0006725 | cellular aromatic compound metabolic process | 149 | 0.002983844 |
| GO:0009132 | nucleoside diphosphate metabolic process | 9 | 0.003731656 |
| GO:0046434 | organophosphate catabolic process | 12 | 0.00647675 |
| GO:0009152 | purine ribonucleotide biosynthetic process | 17 | 0.007972999 |
| GO:0009161 | ribonucleoside monophosphate metabolic process | 19 | 0.008390488 |
| GO:0009185 | ribonucleoside diphosphate metabolic process | 8 | 0.008390488 |
| GO:1901292 | nucleoside phosphate catabolic process | 9 | 0.008390488 |
| GO:1901360 | organic cyclic compound metabolic process | 152 | 0.008646248 |
| GO:0016310 | phosphorylation | 15 | 0.00915045 |
| GO:0006096 | glycolytic process | 7 | 0.009591628 |
| GO:0006165 | nucleoside diphosphate phosphorylation | 7 | 0.009591628 |
| GO:0006757 | ATP generation from ADP | 7 | 0.009591628 |
| GO:0042866 | pyruvate biosynthetic process | 7 | 0.009591628 |
| GO:0046939 | nucleotide phosphorylation | 7 | 0.009591628 |
| GO:0009123 | nucleoside monophosphate metabolic process | 19 | 0.009659855 |
| **Cellular_component** |  |  |  |
| XS11 VS XS11-NO_3_^-^ |  |  |  |
| GO:0005840 | ribosome | 91 | 1.81E-23 |
| GO:0043232 | intracellular non-membrane-bounded organelle | 129 | 1.34E-05 |
| GO:1990904 | ribonucleoprotein complex | 117 | 9.49E-17 |
| GO:0043228 | non-membrane-bounded organelle | 129 | 1.34E-05 |
| XS11 VS XS11-NH_4_^+^ |  |  |  |
| GO:0005730 | nucleolus | 28 | 5.34E-09 |
| GO:0030684 | preribosome | 17 | 2.92E-06 |
| GO:0016021 | integral component of membrane | 397 | 3.04E-06 |
| GO:0031224 | intrinsic component of membrane | 397 | 3.04E-06 |
| GO:0044425 | membrane part | 405 | 1.88E-05 |
| GO:0016020 | membrane | 414 | 4.61E-05 |
| GO:0031981 | nuclear lumen | 45 | 0.001679522 |
| GO:0044428 | nuclear part | 60 | 0.001679522 |
| GO:0005634 | nucleus | 148 | 0.002098148 |
| GO:0032040 | small-subunit processome | 8 | 0.003783903 |
| **Molecular_function** |  |  |  |
| XS11 VS XS11-NO_3_^-^ |  |  |  |
| GO:0003735 | structural constituent of ribosome | 84 | 1.97E-26 |
| GO:0005198 | structural molecule activity | 90 | 3.43E-20 |
| GO:0022804 | active transmembrane transporter activity | 51 | 3.97E-06 |
| GO:0043492 | ATPase activity, coupled to movement of substances | 33 | 0.000837764 |
| GO:0042626 | ATPase activity, coupled to transmembrane movement of substances | 32 | 0.000837764 |
| GO:0015405 | P-P-bond-hydrolysis-driven transmembrane transporter activity | 32 | 0.001026664 |
| GO:0015399 | primary active transmembrane transporter activity | 32 | 0.001026664 |
| XS11 VS XS11-NH_4_^+^ |  |  |  |
| GO:0016491 | oxidoreductase activity | 309 | 3.07E-05 |
| GO:0005375 | copper ion transmembrane transporter activity | 7 | 0.000150455 |
| GO:0046915 | transition metal ion transmembrane transporter activity | 9 | 0.001358388 |
| GO:0015002 | heme-copper terminal oxidase activity | 7 | 0.008390488 |
| GO:0016675 | oxidoreductase activity, acting on a heme group of donors | 7 | 0.008390488 |
| GO:0016676 | oxidoreductase activity, acting on a heme group of donors, oxygen as acceptor | 7 | 0.008390488 |
| GO:0004129 | cytochrome-c oxidase activity | 7 | 0.008390488 |
