# Supplementary material for: Transcriptome Variations in Verticillium dahliae in Response to Two Different Inorganic Nitrogen Sources
Source: Front Microbiol. 2021 Jul 28;12:712701. doi: 10.3389/fmicb.2021.712701 (PMC8355529; doi:10.3389/fmicb.2021.712701)
Supplement: Supplementary Table 2 — Enrichment terms of significantly differentially expressed genes (corrected P-value < 0.05). [file Table_2.DOCX]

**Table S2.** **Enrichment terms of significantly differentially expresse genes (corrected *P* value < 0.05)**

| **Pathway ID** | **Pathway Name** | **Number of DEGs** | **Corrected *P* value** |
| --- | --- | --- | --- |
| XS11 VS XS11-NO_3_^-^ |  |  |  |
| ko03010  ko03020  ko02010 | Ribosome  RNA polymerase  ABC transporters | 87  17  17 | 2.16E-33  0.03134306  0.04177914 |
| XS11 VS XS11-NH_4_^+^ |  |  |  |
| ko00564  ko00565  ko00053  ko00071  ko00280 | Glycerophospholipid metabolism  Ether lipid metabolism  Ascorbate and aldarate metabolism  Fatty acid degradation  Valine, leucine and isoleucine degradation | 33  17  9  21  22 | 0.01624443  0.01624443  0.02203256  0.02203256  0.02203256 |
